# Metabolic model of central carbon and energy metabolisms of growing *Arabidopsis thaliana* in relation to sucrose translocation

**DOI:** 10.1186/s12870-016-0868-3

**Published:** 2016-12-28

**Authors:** Maksim Zakhartsev, Irina Medvedeva, Yury Orlov, Ilya Akberdin, Olga Krebs, Waltraud X. Schulze

**Affiliations:** 1 Department of Plant Systems Biology, University of Hohenheim, Fruwirthstraße 12, 70599 Stuttgart, Germany; 2 Novosibirsk State University, Pirogova 2, 630090 Novosibirsk, Russia; 3 The Federal Research Center Institute of Cytology and Genetics, Russian Academy of Sciences, Lavrentyeva 10, 630090 Novosibirsk, Russia; 4 Biology Department, San Diego State University, San Diego, CA 92182-4614 USA; 5 Heidelberg Institute of Theoretical Sciences, Schloss-Wolfsbrunnenweg 35, 69118 Heidelberg, Germany

**Keywords:** Energy metabolism, Multi-compartment metabolic model, Central carbon metabolism, Sucrose metabolism, Sucrose transport, Flux balance analysis, Diurnal growth

## Abstract

**Background:**

Sucrose translocation between plant tissues is crucial for growth, development and reproduction of plants. Systemic analysis of these metabolic and underlying regulatory processes allow a detailed understanding of carbon distribution within the plant and the formation of associated phenotypic traits. Sucrose translocation from ‘source’ tissues (e.g. mesophyll) to ‘sink’ tissues (e.g. root) is tightly bound to the proton gradient across the membranes. The plant sucrose transporters are grouped into efflux exporters (SWEET family) and proton-symport importers (SUC, STP families). To better understand regulation of sucrose export from source tissues and sucrose import into sink tissues, there is a need for a metabolic model that takes in account the tissue organisation of *Arabidopsis thaliana* with corresponding metabolic specificities of respective tissues in terms of sucrose and proton production/utilization. An ability of the model to operate under different light modes (‘light’ and ‘dark’) and correspondingly in different energy producing modes is particularly important in understanding regulatory modules.

**Results:**

Here, we describe a multi-compartmental model consisting of a mesophyll cell with plastid and mitochondrion, a phloem cell, as well as a root cell with mitochondrion. In this model, the phloem was considered as a non-growing transport compartment, the mesophyll compartment was considered as both autotrophic (growing on CO_2_ under light) and heterotrophic (growing on starch in darkness), and the root was always considered as heterotrophic tissue dependent on sucrose supply from the mesophyll compartment. In total, the model includes 413 balanced compounds interconnected by 400 transformers. The structured metabolic model accounts for central carbon metabolism, photosynthesis, photorespiration, carbohydrate metabolism, energy and redox metabolisms, proton metabolism, biomass growth, nutrients uptake, proton gradient generation and sucrose translocation between tissues. Biochemical processes in the model were associated with gene-products (742 ORFs). Flux Balance Analysis (FBA) of the model resulted in balanced carbon, nitrogen, proton, energy and redox states under both light and dark conditions. The main H^+^-fluxes were reconstructed and their directions matched with proton-dependent sucrose translocation from ‘source’ to ‘sink’ under any light condition.

**Conclusions:**

The model quantified the translocation of sucrose between plant tissues in association with an integral balance of protons, which in turn is defined by operational modes of the energy metabolism.

**Electronic supplementary material:**

The online version of this article (doi:10.1186/s12870-016-0868-3) contains supplementary material, which is available to authorized users.

## Background

### Aim

The aim of this research was to build a multi-compartmental metabolic model of growing *Arabidopsis thaliana.* The integrated model should describe biomass growth both in light and in dark phases with corresponding formation and consumption of starch and sucrose. Furthermore, the structured metabolic model should take into account major pathways of primary metabolism such as sugar metabolism, central carbon metabolisms, photosynthesis, photorespiration, energy and redox metabolism, proton turnover, sucrose translocation from source to sink tissues and biomass growth. In future, we will use the model to recognize cause-effect relationships and describe regulatory processes in carbon metabolism and transport.

### Biological background

In growing plants, sucrose is the most widespread sugar used to supply both carbon and energy from ‘source’ tissues (e.g. autotrophic mesophyll) to ‘sink’ tissues (e.g. heterotrophic roots, growing shoots or reproductive organs) to build up a biomass [1]. During photosynthesis in plastids of mesophyll cells, triose phosphates (GAP, DHAP) are synthesised and exported into the cytoplasm to support formation of sucrose and biomass. During growth in the light, starch is formed and accumulated in the plastids and becomes a part of the biomass [2, 3]. Starch is a repository of carbon which is later used during the dark phase as the primary carbon source for biomass formation [2] and fuelling of sucrose biosynthesis and its transport. The diurnal dynamics of starch accumulation is generally well documented in plants, and particularly in *Arabidopsis thaliana* [2] this process was even subjected to the analysis of regulatory patterns by means of dynamic mathematical modelling [4]. Perturbation of these tightly regulated metabolic processes results in growth phenotypes of the plants. For example, disruption of the plant’s ability to invest carbon into the day-time storage of starch in the *pgm* mutant [5] results in higher cytosolic sucrose levels, higher respiration rates, retarded growth [6], low seed yield [7], and slow root growth at night [8].

**Fig. 1:**
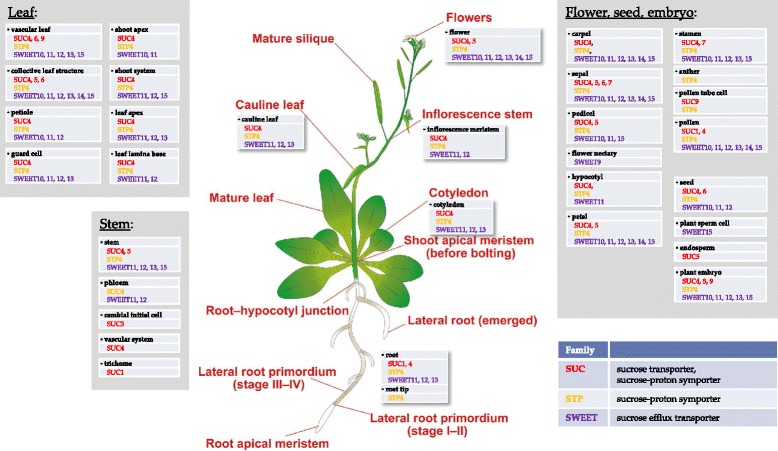
Expression of sucrose transporter genes in different tissues of *Arabidopsis thaliana*. Overview of the sucrose transporter families *SWEET* (sucrose efflux transporters), *SUC* and *STP* (sucrose-proton symporters). The image of *Arabidopsis* has been adopted from [83]

Sucrose is translocated within the phloem, which is loaded in source tissues and unloaded in sink tissues [9, 10]. Loading/unloading goes through both symplastic and apoplastic structures. The symplastic transport mechanism does not require any specific sucrose carriers and relies on plasmodesmal connection of cells. The apoplastic sucrose transport mechanism involves several efflux/influx carriers and translocation of sucrose across membranes [9–11]. Thereby,sucrose efflux from source cells follows its concentration gradient and influx of sucrose into recipient (or sink) tissue happens in symport with protons along their concentration gradient. The proton gradient across the membrane in turn is actively formed by the plasma membrane H^+^-ATPase activity [10, 12]. There are three families of sucrose transporters known in *Arabidopsis thaliana*: *SWEET*, *SUC* and *STP*. The families of sucrose transporters differ in functional properties: members of the *SWEET* family facilitates sucrose efflux [13], whereas members of the *SUC* and *STP* families perform sucrose or sugar uptake in symport with protons [14–16]. Sucrose-proton symporters display wide variety in their affinities to sucrose. For example, *SUC2* is a high-affinity, while *SUC4* is low-affinity sucrose-proton symporters [17]. Knock-out mutants of the sucrose transporters have characteristic phenotypes [18]: Mutants of *SUC2,* which is the major transporter involved in phloem loading of sucrose, have even a lethal phenotype under sucrose-free growth conditions, and mutants of sucrose symporter *SUC1* were shown to be important in pollen development and pollen tube growth [19]. Mutants of the sucrose exporters *SWEET11/12* [20] show particularly stunted root growth on sucrose-free medium and they accumulate starch in the leaves. All plant tissues simultaneously express efflux (*SWEET*) and influx (*SUC*, *STP*) transporters (Fig. 1), which points to coupling of efflux and influx mechanism during sucrose translocation from cell to cell and the readiness of almost all plant tissues to exchange sucrose between each other depending on the current needs.

**Fig. 2:**
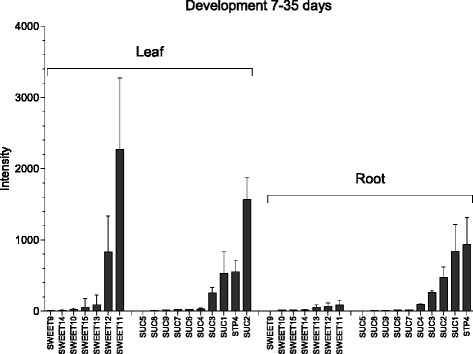
Expression of sucrose transporter genes in *Arabidopsis thaliana* in leaves and roots during development. Absolute intensity values of sucrose transporter genes expression in leaves and roots during development (7–35 days). SWEET efflux-transporters and SUC/STP influx-transporters are both highly expressed in leaf while the root mainly expresses SUC/STP influx-transporters. The plot is based on gcRMA normalized data selected from [84] based on TAIR ExpressionSet 1007966126 [85]

**Fig. 3:**
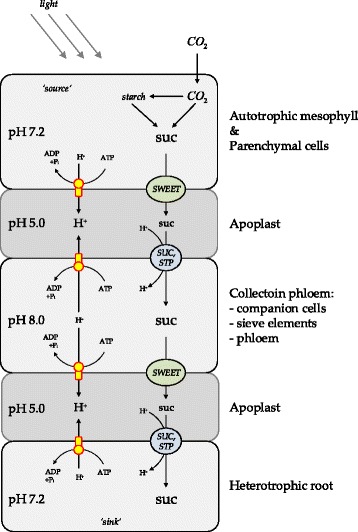
Simplified mechanism of sucrose translocation from autotrophic to heterotrophic tissues via connecting tissue. The autotrophic tissue (mesophyll) synthesises sucrose that is translocated to heterotrophic tissue (root) as carbon and energy source to build biomass. Metabolically active tissues form a proton gradient with the extracellular space (apoplast), which is used by the sink tissue to uptake sucrose. *suc* – sucrose, *H* ^+^ – proton, *SWEET* – sucrose efflux transporters, *SUC*,*STP* – sucrose-proton symporters. Size of letters represents relative concentrations

Expression analysis [21] of sucrose transporter genes in leaves and roots revealed particularly high expression of SWEET11,12 in autotrophic mesophyll tissue, whereas expression level of sucrose-proton symporters *SUC1,2* and *STP4* dominate in heterotrophic root (Fig. 2). Based on the understanding of the existence of the net-flux of sucrose directed from a leaf as the source tissue to a root as the sink tissue during growth of a plant [9, 10], it is valid to generalize the molecular mechanism of sucrose translocation among tissues (Fig. 3). Such generalized view on the molecular mechanisms of sucrose translocation takes in account only two chemical motive forces (sucrose and proton gradients) and respective transporters that use them (*SWEET* efflux transporters and *SUC*/*STP* sucrose-proton symporters).

In most sink tissues, sucrose is primarily used as a carbon source to support growth and build up biomass. However, sucrose can also serve as metabolite invested into storage compounds in root tissues. It can be converted to starch as in potato tubers or it can directly be stored in the vacuole as in sugar beet or sugar cane. Thus, the whole plant growth is tightly dependent on regulation of sucrose metabolism and transport. The cause-effect relationship of sucrose transport between tissues and phenotypic traits of plants is an important area of current plant research [22].

### Mathematical background

The analysis of metabolic networks with a rational approach is an efficient tool for engineering of plant systems [23, 24]. Model-based approaches are increasingly used also in plant biology to gain functional insights and even prediction of metabolic processes [25, 26]. In general, network modelling involves several stages: (i) collection of a priori knowledge and construction of the model by network reconstruction [27, 28]; (ii) a priori check of the proposed metabolic network model, such as topological analysis of stoichiometric matrix to eliminate structural gaps; (iii) addition of constraints and formulation of objective function to the model; (iv) network optimization using constraint-based analyses such as flux balance analysis (FBA); (v) *a posteriori* consistency check of the identified model by metabolic flux analysis (MFA); (vi) validation of the model with a newly generated experimental dataset [29, 30]. Based on this workflow the successful metabolic models of *Arabidopsis thaliana* of different scales were already suggested [23, 25, 28, 31–34] including multi-tissue genome scale metabolic models [32, 35, 36]. However, each of these existing metabolic models is dedicated to a specific biological phenomenon to be better understood, such as photosynthesis [37] or the Calvin-Benson cycle [38]. On a more global scale, the metabolic costs for all amino acids and proteins in a given network were calculated based on the flux balance analysis of genome-scale metabolic network of *Arabidopsis thaliana* in light and dark conditions [39]. Flux Balance Analysis combined with turnover measurements of ^13^C-labeled metabolites can provide deeper insights into the underlying metabolic processes. Stability of metabolic fluxes in central metabolism of *Arabidopsis thaliana* root cells was tested against environmental variation of oxygen using such approach [40, 41].

The stoichiometric models can in future be further developed into a dynamic model based on kinetic expressions of particular biochemical reactions and their integration [4, 37, 42, 43]. Kinetic modelling of plant metabolism was used to unravel local and global system features, such as flux and concentration control coefficients and regulation patterns [44] in relation to different external or internal states, stimuli and conditions [31]. A kinetic model of sugar metabolism throughout tomato *Solanum lycopersicum* fruit development revealed importance of different enzymes on different development stages, as well as importance of sugar accumulation in vacuole together with organic acids to enable osmotic-driven vacuole expansion during the cell division [45]. Also, different regulatory scenarios of starch turnover by the circadian clock through dynamic adjustment of starch turnover to changing environmental conditions were suggested based on mathematical modelling [4].

**Fig. 4:**
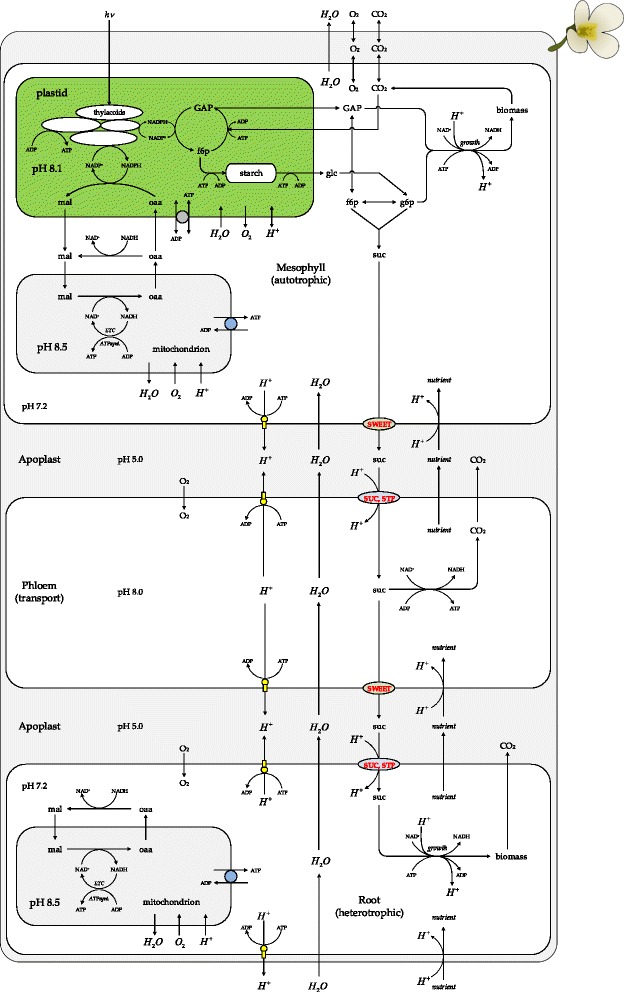
Schematic circuit of the central carbon and energy metabolisms of *Arabidopsis thaliana*. The model consists of super-compartment ‘plant’, which includes growing autotrophic sub-compartment ‘mesophyll’, non-growing transport sub-compartment ‘phloem’ and growing heterotrophic sub-compartment ‘root’. The inner space of the super-compartment ‘plant’ was defined as of ‘apoplast’. The ‘mesophyll’ compartment contained ‘plastid’ and ‘mitochondrion’ while the ‘root’ compartment only contained ‘mitochondrion’. Details of metabolic pathways were hidden in order to focus only on the specificity of the sucrose synthesis/translocation in relation of H^+^-turnover, nutrient and water transport between tissues. *hv* – light photon; *GAP* – glyceraldehyde 3-phosphate; *suc* – sucrose; *g*6*p* – glucose-6-phosphate; *f*6*p* – fructose-6-phosphate; *oaa* – oxaloacetate; *mal* – malate; *H* ^+^ – proton; ETC. – electron transport chain, that performs oxidative phosphorylation; *growth* – collective set of reactions resulted in formation of biomass; *ATPsunt.* – ATP synthase; *nutrient* – nutrients such as *NO*_3_^−^, *HPO*_4_^2 −^, *SO*_4_^2−^, *SWEET* – sucrose efflux transporters*, SUC,STP* – sucrose-proton symporters

In this study, we used this principal molecular mechanism of sucrose translocation as the basis for a multi-compartmental metabolic model (Fig. 4). Thus, the quantification of proton balance in the source, phloem and sink tissues is required for the quantitative assessment of sucrose translocation. Therefore, the integration of basic metabolic processes involved in the production/utilization of protons in these tissues is additionally required.

### Major findings

The multi-compartmental metabolic network of *Arabidopsis thaliana* was reconstructed and optimized in order to explain growth stoichiometry of the plant both in light and in dark conditions. Balances and turnover of energy (ATP/ADP) and redox (NAD(P)H/NAD(P)) metabolites as well as proton in different compartments were estimated. The model showed that in light conditions, the plastid ATP balance depended on the relationship between fluxes through photorespiration and photosynthesis including both cyclic and non-cyclic electron flow. The ATP balance in plastid depended on the ratio between these processes, and therefore can be either deficient, self-supported or producing a surplus. The excess of redox potential from the photosynthetic light system was translocated to the mitochondrion via the malate/oxaloacetate shuttle. The model showed that the mitochondrion consumed protons under both ‘light’ and ‘dark’ conditions and provided ATP to the cytoplasm. Matching the proton fluxes with proton-dependent translocation of sucrose between tissues from source to sink in light and dark conditions corresponded well to the known molecular mechanism of sucrose transport.

## Results and discussion

### Model formulation

In order to model the interconnection of sucrose metabolism and its proton-dependent transport between different tissues in a multi-compartment metabolic model it was necessary firstly to account for all major proton releasing/utilizing cellular metabolic processes related to biomass formation in order to reconstruct proton fluxes, and compare them with known macroscopic exchange processes (Table 1). Secondly, it was necessary to model contributions of photosynthesis with concomitant water photolysis and aerobic respiration with concomitant water formation into energy and proton balances and correspondingly into formation of the proton motive force between tissues. Furthermore, energy- and redox-metabolisms (photosynthesis, photorespiration, aerobic respiration and glycolysis) had to be modelled in connection with the central carbon metabolism. We defined two possible carbon sources (i.e. CO_2_ and starch) for biomass formation dependent on the energy metabolism mode when photosynthesis is either on or off. CO_2_ was used as carbon source in the photosynthetic growth phase (light) and starch was used in the respiratory growth phase (dark). Finally, we had to ensure constant distribution of sucrose between tissues with retaining the corresponding direction of sucrose translocation under both growth conditions.

**Table 1:** Generally accepted directions of macroscopic metabolic fluxes in ‘light’ and ‘dark’ growth phases of Arabidopsis thaliana

Flux	Light	Dark	Reference
hv (light photons)	*−*	0	
CO_2_	*−*	+	[1]
O_2_	+	*−*	[1]
H_2_O	*−*	+	[1]
H^+^	*−*	*−*	Experimental
Starch	+	*−*	[2, 4]
Biomass^a^	+	+	

‘0’ absence; ‘*−*‘consumption or utilization; ‘+’ production or formation; ^a^ the biomass formation assumes continuous consumption of nitrogen, phosphorus and sulphur sources

In our model, we simplified the complex plant tissue organization to four principal compartments: (i) the super-compartment ‘plant’, which includes (ii) a growing autotrophic ‘mesophyll’, (iii) a heterotrophic growing ‘root’ and (iv) a non-growing ‘phloem’ compartment. Mesophyll, phloem and root were interconnected through inner space of the super-compartment ‘plant’, which played the functional role of ‘apoplast’ (Figs. 3 and 4). To model the exchange of solutes with the external environment, the ‘root’ was set to acquire water, protons, nutrients and transport them up to the ‘mesophyll’ via the ‘phloem’, while the ‘mesophyll’ was exchanging CO_2_, O_2_, excreting an excess of water, and providing sucrose in counter-flux to the ‘root’ via the ‘phloem’ (Fig. 4). The ‘mesophyll‘compartment included sub-compartments: plastid and mitochondrion, while the ‘root’ compartment contained only mitochondria. Additionally, the model included a set of reactions that imitated the vacuole, as a virtual sink (only accumulating) compartment. We assigned accumulation of nitrogen, orthophosphate, sulphur, and carbon in form of ash to the virtual vacuole. In addition, since sucrose reserves were also defined as a biomass constituent, therefore sucrose contributing to growth was also irreversibly stored in the virtual vacuole.

The network was formulated to focus only on the major relevant metabolic processes from the central carbon metabolism that contribute to the outlined phenomena of sucrose formation, translocation and degradation along with proton formation in metabolism and energy/redox reactions. The elemental and charge balances of each metabolic reaction in the model were verified based on elemental composition of compounds and their known charges.

The model was developed in an iterative way of (i) network reconstruction, formulation, correction, (ii) network setup analysis (to remove inconsistencies), (iii) topological analysis (to remove structural gaps and false-positive effects), and (iv) Flux Balance Analysis to optimize objective function and reach biological consistency. The outcomes of the predicted macroscopic fluxes were compared with generally accepted directions of macroscopic input/output fluxes of the plant metabolites, which are well documented for ‘light’ and ‘dark’ growth phases (Table 1). Additionally, directions of intracellular fluxes predicted by FBA were checked for the biological consistency and in case of inconsistency or biological irrelevance, the network setup was re-designed in order to eliminate these inconsistencies. Then cycle of the analyses was repeated until the model reflected the biologically relevant picture.

#### Proton transport

The main uptake path of proton from environment to the phloem cell in the model was in symport with nutrients such as orthophosphates, nitrates, or sulphates (Fig. 4). Nevertheless, there is also ATP dependent efflux of protons from cells through the activity of the plasma membrane H^+^-ATPase in a stoichiometric ratio 1:1 [46]. Thus, the model takes in account simultaneous and independent influx/efflux of protons, but with overall net-flux of protons from the environment to the plant cells as determined experimentally from measured alkalinisation of the growth media for growing *Arabidopsis* roots. Under standard growth conditions in hydroponic setup [47], the pH value of the ½MS-medium was found to increase from 5.7 to 6.2 over a period of two weeks.

#### Photosynthesis

Special attention was paid to modelling of energy metabolism and photosynthetic light reactions. The photosynthesis light reactions were formulated in accordance with descriptions in AraCyc [48] and an existing model [37]. There are two processes that can significantly influence the energetic efficiency of photosynthesis, namely (i) photorespiration (oxidative photosynthetic carbon cycle) [49] and (ii) cyclic electron flow through the photosynthesis light reactions [50, 51]. Photorespiration: Although in some cases photorespiration is considered as a ‘security valve’ to reduce the consequences of over-oxygenation in plastids, it is nevertheless considered as the essential part of the photosynthesis [52], which potentially reduces photosynthetic output by 20–30 % in C3 plants [53]. Some reactions of photorespiration occur in peroxisome and mitochondrion, however to avoid complication of the model with introduction of an additional compartment (i.e. peroxisome) and introduction of additional pathways to mitochondria, the corresponding peroxisomal reactions were written within the cytoplasm of the mesophyll. In this way, photorespiration was connected to the cytoplasmic folate metabolism. Cyclic electron transport (CET): The cyclic electron transport was reported to occur mainly at high irradiances in combination with very low CO_2_ concentration [54], nevertheless the CET generally is reported as an essential element of the photosynthesis system [54–56]. Thus, the current version of the model includes photosynthesis with both non-cyclic and cyclic electron transports through photosynthetic light reactions and photorespiration. The proton motive force for ATP synthase in plastid was formed by the cytochrome *b*_*6*_ */f* complex and the proton/ATP stoichiometry of ATP synthase was fixed to 4.0 [57, 58].

Plastid metabolism was restricted to photosynthetic light reactions, Calvin-Benson cycle (CBC), pentose-phosphate pathway (PPP), sulphate and nitrite reduction pathways, synthesis/degradation of starch. Sulphate and nitrite reduction pathways were interconnected through conserved moiety of ferredoxin(red)/ ferredoxin(ox) with photosynthetic light reactions, as well as to NADPH/NADP^+^ conserved moieties. Under light conditions, reduced ferredoxin was mainly consumed by ferredoxin-NADP^+^ oxidoreductase (FNR), which provided NADPH for the Calvin-Benson cycle, whereas the oxidative part of PPP was inactive. However, under dark conditions, when photosynthesis light reactions were inactive, the oxidative part of PPP became active and, in the model, it was considered as the only provider of NADPH in plastids during darkness. Formed NADPH was used by FNR to reduce ferredoxin and therefore to feed sulphate and nitrite reduction during dark phase.

We did not consider other central carbon metabolic pathways in plastid (fatty acids synthesis, amino acids and glutathione biosynthesis), due to increasing complexity of the model and since these pathways did not contribute to a better reflection of the molecular relationships of the phenomenon under study. Thus, the elaborated pathways considered in the plastid were just enough to model photosynthesis, energy and redox balance, proton balance, CO_2_ fixation, triose phosphate synthesis as well as starch synthesis and degradation.

#### Starch metabolism

During the light-growth-phase, starch was synthesized and stored as biomass constituent. During the dark-growth-phase it became the only carbon and energy source. The model considered its consumption as a virtually external metabolite.

#### Folate (or C1) metabolism

The tetrahydrofolate transformations are important part of C1-metabolism [59]. This pathway connected the transfer of C1-group with amino acid metabolism, nitrogen metabolism [59], and with photorespiration when it was active.

#### Nitrogen and sulphate metabolism

In the mesophyll, nitrate was reduced to nitrite in the cytoplasm and then transported to the plastid for further reduction to glutamate/glutamine, which were returned back to the cytoplasm, and further used in transamination reactions for synthesis of others amino acids. Sulphate was transported to the plastid, where it was reduced to H_2_S, which was returned back to cytoplasm and used further to synthesize cysteine [60]. Nitrate and sulphate reduction are metabolic processes that demand reduced equivalents and overall utilize free protons [7]. Therefore, these reactions were additional redox-loads for the plastidic redox-balance. In roots, the nitrogen and sulphate metabolism was modelled in the cytoplasm and were fed by NADPH produced by the oxidative part of cytoplasmic PPP. This was a compromise to avoid modelling amyloplasts in root [61, 62].

To ensure functional metabolite exchange of metabolites and redistribution of energy and redox load between organelles (plastid, mitochondrion) and cytoplasm so-called metabolite translocators and shuttles were included in the model. It is well documented that the energy-, redox-, sulphur-, nitrogen- and carbon- metabolisms are interdependent via such exchange processes [12, 61, 63]. We integrated the following exchange processes in the model: ATP/ADP translocation among plastid/cytoplasm/mitochondrion [63], redox exchange via malate/oxaloacetate shuttle (dicarboxylate translocators) between plastid/cytoplasm/mitochondrion [64, 65], carbon (DHAP, GAP) exchange (triose phosphate/phosphate translocator) between plastid/cytoplasm [61, 63, 66], amino acids and ammonia exchange between plastid/cytoplasm [61, 63, 65], organic acid exchange between cytoplasm/mitochondrion [1, 65].

**Fig. 5:**
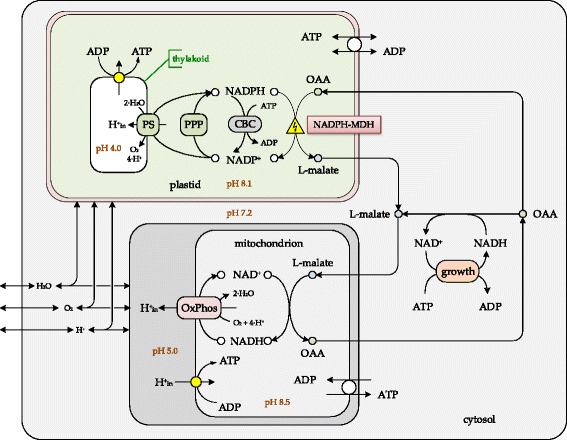
Generalized view on functioning of the malate/oxaloacetate shuttle in the mesophyll. The depicted metabolic scenario was elaborated based on the Flux Balance Analysis. PS – photosynthesis system; PPP – pentose-phosphate pathway; CBC – Calvin-Benson cycle; NADPH-MDH –NADPH-dependent malate dehydrogenase, which is marked as light sensitive; OAA – oxaloacetate; OxPhos – oxidative phosphorylation; ATP/ADP translocator is bidirectional in plastid and unidirectional in mitochondrion

Such metabolic architecture provided higher metabolic plasticity. For example, implementation of the malate/oxaloacetate shuttle avoided over-reduction of the plastid during the light-growth-phase [63] by translocating redox equivalent from plastid to the mitochondrion, where it was used for ATP generation (Fig. 5). Special attention was paid to the triose phosphate/phosphate translocator (TPT) that exchanges triose phosphates (GAP, DHAP) between plastid and cytoplasm in antiport with orthophosphate [61, 63, 66–68]. This exchange process controls the activity of photosynthetic ATP formation in the plastid [12, 68]. Thus, control of orthophosphate transport into the plastid exerted control on whether triose phosphates were exported to the cytoplasm or used in the plastid for starch synthesis. Since GAP and DHAP can be interconverted by near equilibrium enzyme triose phosphate isomerase (TPI: EC 5.3.1.1), in the model only GAP was subjected to reversible exchange between plastid and cytoplasm in order to avoid introduction of an additional parallel route.

The final version of the model (Additional file 1: Figure S1) was composed of 400 transformers, among which were 229 metabolic reactions from 41 different metabolic pathways, 155 transporters and 16 polymerization steps. The transformers connected 423 compounds, among which 413 were balanced compounds. In the model, external metabolites such as gases, nutrients, biomass were unbalanced compounds, because they were considered as being an infinite source or sink. All biochemical reactions were associated with 742 genes, whose products performed the corresponding biochemical reactions.

### Topological analysis

The model in total had 15 degrees of freedom, with inner degree of freedom equal to 10 and outer degree of freedom equal to 5 (Table 2). The inner degree of freedom 10 was due to the presence of ten parallel routes in the network structure. All parallel routes involved sub-sets of reactions that create cyclic structures in the network and all of them related to energy metabolism in the plastid, cytoplasm and mitochondrion, where cyclic pathways such as Calvin-Benson cycle, TCA cycle, photorespiration, or the malate/oxaloacetate shuttle, occur naturally.

**Table 2:** Metrics and topological indicators of the stoichiometric model of Arabidopsis thaliana

Entity	Value	
Content:	Transformers:	400
	Reactions:	229
	Transporters:	155
	Polymerizators:	16
	Compounds:	423
	Balanced compounds:	413
	ORFs:	742
Degree of freedom (*df*)	Total *df*:	15
	Inner *df*:	10
	Outer *df*:	5
Conserved moieties	There are 28 conserved moieties across all compartments of the model, among them there are only a biologically determined moieties: - NAD(P)H/NAD(P) [all compartments] - CoQH2/CoQ [mitochondria] - THF/5-Formyl-THF/5,10-Methylene-THF/5-Methyl-THF [tetrahydrofolate : cytoplasms] - CoA/Acetyl-CoA [CoA : cytoplasms] - CoA/Acetyl-CoA/Succinyl-CoA [CoA : mitochondria] - CytC(red)/ CytC(ox) [cytochrome c : mitochondria] - Fd(red)/Fd(ox) [ferredoxin : plastid] - PQH2/PQ [plastoquinone : plastid] - PC(red)/PC(ox) [plastocyanine : plastid] - ATP/ADP [mitochondria] - ADP-glc/ATP/ADP/AMP/APS [plastid]	
Nullspace	15 solution spaces	
Parallel routes & cycles	10 routes	

Analysis of conserved moieties in all compartments revealed only those with biological relevance (Table 2). Interestingly, conserved moiety ATP/ADP/AMP was not in the list of cytoplasmic conserved moieties unlike in the plastid, since cytoplasmic AMP is *de novo* synthesized and interconnected to the ATP/ADP pool via adenylate kinase, and, at the same time in the model, AMP was a monomer for RNA/DNA polymerization. Thus, ATP/ADP/AMP was an open moiety in the cytoplasm.

Determination of the nullspace of a stoichiometric matrix of a model is an important topological criterion to assess the feasible space containing all possible solutions of the linear equations **S** × **v** = **0** without taking into account thermodynamic irreversibility. Thus, the nullspace analysis is used to reveal non-functional regions of the network by finding solution spaces of all sub-networks that are able to operate at steady state. In the model there were 15 overlapping sub-networks that all together cover all the compounds and reactions in the suggested network.

### Flux Balance Analysis (FBA)

The structure of the metabolic network and exchange processes between compartments and sub-compartments were optimized using FBA. The FBA of the *Arabidopsis thaliana* model was solved using two sets of constraints that were applied to the single network. Each set of constraints mimicked either ‘light’ or ‘dark’ conditions (Table 3). For the light condition, the impact of different ratios between photorespiration and photosynthesis on plastid energy metabolism, and the contribution of different ratios between cyclic and non-cyclic electron flow through photosynthesis light reactions were explored. Respective constraints are formulated in Table 3. Derived stoichiometric coefficients were normalized per biomass [*mol i*/*mol X*]. As a result, overall growth stoichiometry (Table 3) matches the macroscopic input/output fluxes outlined in Table 1 for light and dark growth scenarios.

**Table 3:** Table of flux constraints used for Flux Balance Analysis (FBA) of the stoichiometric model of *Arabidopsis thaliana*

Growth conditions	Objective function	Constraints	Interpretation of the constraints
‘Light’	*max* (T.Biomass.ext)	T.Biomass.ext < = 5000 SK = 0 0.25*RPC_plastide - RPC2_plastide = 0 T.starch.ext = 0 T.CO2.ext = 0 T.CO2.ext_rev > = 0 -----*add-on*----- FQR = 0 *or* T.ADP.plastid - T.ATP.plastid = 0 *or* (0 … 0.5)*FNR - FQR = 0 *or* RPC2_plastide = 0 GLYK = 0	Maximization of biomass formation under light assumes that: • A plant consumes CO_2_ as the carbon source (T.CO2.ext = 0, T.CO2.ext_rev > = 0) there are two constraints because CO_2_ transport is reversible • All formed starch becomes a part of the biomass (T.Starch.ext = 0) • There is no starch degradation under light and therefore starch kinase is inactive (SK = 0) • Photorespiration flux was fixed at 20 % of flux through RuBisCo (0.25*RPC_plastide - RPC2_plastide = 0) at all tested conditions Additionally, the impact of different degree of cyclic electron flow through photosynthesis light reactions (particularly through ferredoxin-plastoquinone reductase; FQR) was estimated by means of add-on constraints: • Either … cyclic electron flow is inactive (FQR = 0) • Or … flux through ATP/ADP translocator between plastid and cytoplasm is inactive (T.ADP.plastid – T.ATP.plastid = 0), thus plastid’s ATP balance becomes self-sufficient • Or … flux through FQR varies relative to the non-cyclic electron flow through ferredoxin-NADP^+^-oxidoreductase (FNR) ((0 … 0.5)*FNR – FQR = 0) • Or … flux through photorespiration pathway is zero (RPC2_plastide = 0; GLYK = 0), but cyclic electron flow through FQR is subjected to optimization
Resulted growth stoichiometry for ‘light’ conditions (the stoichiometric coefficients [*mol i*/*mol X*] are normalized per biomass):			
[PR^*^ = 0.25; FQR = 0]: [PR = 0.25; FQR/FNR = 0.37]: [PR = 0.25; FQR/FNR = 0.5]: [PR = 0; FQR/FNR = 0.1]:	3.26e7 × *CO*_2_ + 2.12e7 × *H*_2_ *O* + 1.00e6 × *HPO*_4_^2 −^ + 1.99e4 × *SO*_4_^2 −^ + 1.52e6 × *H* ^+^ + 5.04e6 × *NO*_3_^−^ + 5.26e8 × hv = > 3.98e7 × *O*_2_ + Biomass_plant 3.26e7 × *CO*_2_ + 2.12e7 × *H*_2_ *O* + 1.00e6 × *HPO*_4_^2 −^ + 1.99e4 × *SO*_4_^2 −^ + 1.52e6 × *H* ^+^ + 5.04e6 × *NO*_3_^−^ + 5.58e8 × hv = > 3.98e7 × *O*_2_ + Biomass_plant 3.26e7 × *CO*_2_ + 2.12e7 × *H*_2_ *O* + 1.00e6 × *HPO*_4_^2 −^ + 1.99e4 × *SO*_4_^2 −^ + 1.08e6 × *H* ^+^ + 5.04e6 × *NO*_3_^−^ + 5.92e8 × hv = > 3.98e7 × *O*_2_ + Biomass_plant 3.26e7 × *CO*_2_ + 2.12e7 × *H*_2_ *O* + 1.00e6 × *HPO*_4_^2 −^ + 1.99e4 × *SO*_4_^2 −^ + 5.84e6 × *H* ^+^ + 5.04e6 *NO*_3_^−^ × + 3.90e8 × hv = > 3.98e7 × *O*_2_ + Biomass_plant		
‘Dark’	*max*(T.Biomass.ext)	T.Biomass.ext < = 5000 PGM3_plastid = 0 RPC_plastide = 0 RPC2_plastide = 0 GLYK = 0 GDC = 0 T.starch.ext > = 0 T.hv.ext = 0	Maximization of the biomass formation in darkness assumes that: • There is no light (T.hv.ext = 0) • Therefore RuBisCo is inactive (RPC_plastide = 0) • Correspondingly photorespiration is also inactive (RPC2_plastide = 0, GLYK = 0, GDC = 0) • Consequently there is no synthesis of new starch, therefore phosphoglucomutase is inactive (PGM3_plastid = 0) • Starch is the carbon source for biomass formation (T.Starch.ext > = 0), which previously has been deposited in course of the light phase
Resulted growth stoichiometry for ‘dark’ conditions (the stoichiometric coefficients [*mol i*/*mol X*] are normalized per biomass): 8.48e6 × *O*_2_ + 1.00e6 *HPO*_4_^2 −^ × + 1.99e4 × *SO*_4_^2 −^ + 3.84e6 × *H* ^+^+ 5.04e6 × *NO*_3_^−^ + 1.34e4 × Starch = > 1.55e7 × *CO*_2_ + 1.92e7 × *H*_2_ *O* + Biomass_plant			

*PR – photorespiration; constrain PR = 0.25 is the ratio between flux through photorespiration and photosynthesis, particularly through RuBisCo in Calvin-Benson cycle

Modelling of the metabolic activity of the mesophyll was the most complex task due to light-dependent activity of energy-, redox- and carbon-metabolism in plastid, in terms of CO_2_ and O_2_ consumption/production, starch formation/degradation, triose phosphate export/import, or under conditions of photorespiration on/off. The proton balance in the mesophyll was especially influence by energy and redox metabolism particularly water photolysis and oxygen reduction (Figs. 4 and 5). In contrast, the root metabolism was almost invariant under light and dark growth condition, being the constant sink for sucrose.

Chosen stoichiometry of the anabolic reactions leading to the biomass formation predicted an elemental composition of the *Arabidopsis thaliana* biomass as CH_1.592_O_0.834_N_0.144_P_0.033_ (*MW*_*x*_ = 29.88 [*g*_*dw*_/*Cmol*]), which only slightly differed from averaged elemental biomass compositions of microbial species (CH_1.596_O_0.396_N_0.216_P_0.017_; *MW*_*x*_ = 24.59 [*g*_*dw*_/*Cmol*]) [69–72]. We cannot judge about significance of the observed differences, since there is no confident information published on elemental composition of *Arabidopsis thaliana*.

### ATP and NAD(P)H balances in mesophyll

While interpreting of the FBA results, main attention was placed on the metabolic activity of the mesophyll, since in the model the mesophyll constituted 85 % (w/w) of the mass fraction within the plant biomass.

#### Plastid

Photosynthetic light reactions (PLR) in the plastid were modelled in details in order to provide maximal plasticity in ATP and NADPH allocations. It is well documented, that fixation of CO_2_ to yield triose phosphate (GAP or DHAP) in the Calvin-Benson cycle requires a maximal theoretical ATP/NADPH ratio of 1.5 [1, 63]. However, non-cyclic photosynthetic electron transport (in the way as it is written in AraCyc [48]) provides an ATP/NADPH ratio of 1.0 and experimentally measured values range up to 1.3 [73]. Thus, the demand for ATP in plastid can exceed the level of ATP synthesis provided by non-cyclic electron transport in photosynthetic light reactions. To overcome this limitation, the C3 plants use several pathways to adjust the ATP/NADPH ratio in the plastid stroma and to finally ensure CO_2_-fixation in the Calvin-Benson cycle: (i) increased cyclic electron transfer in photosynthetic light reactions to increase ATP yield without concomitant NADPH yield [55]; (ii) increased ATP consumption through photorespiration; (iii) importing/exporting ATP from/to cytoplasm via ATP/ADP translocator [61, 63]; (iv) NADPH consumption in reductive biosynthetic pathways in the plastid stroma such as nitrite- and sulphate-reduction [60]; (v) shuttling of reduced equivalents from plastid to mitochondrion via the malate/oxaloacetate shuttle [64] (Fig. 5); (vi) shuttling of ATP and reduced equivalents to the cytoplasm through DHAP/GAP shuttle [63] (which was not included into the model, for the reasoning see above).

To explore the contributions of all pathways to plastid ATP/NADPH balance, the FBA was correspondingly constrained in a series of independent runs (Table 3). Growth in the light meant that CO_2_ was the carbon source, photosynthesis (including both cyclic and non-cyclic electron flow) and also photorespiration were active, NADPH was generated by photosynthesis, ATP was generated by the plastidic ATP-synthase, but dependent on the actual ATP/NADPH balance it was additionally imported/exported from/to cytoplasm, triosephosphates were formed in the Calvin-Benson cycle and used both for export to cytoplasm and starch synthesis. Growth in the darkness meant that carbon source was starch and in plastid the photosystem I and II, RuBisCO, and therefore photorespiration were inactive, ATP was completely imported from the cytosol and NADPH was generated by partially active Calvin-Benson cycle and PPP fed by triosephosphates obtained from starch degradation.

**Fig. 6:**
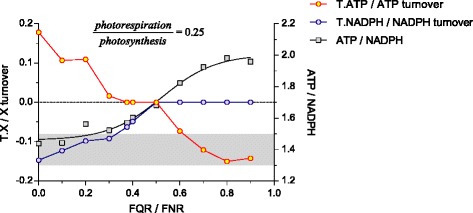
Relationship between translocated fractions of ATP and NADPH within their total balance in the plastid under light conditions. Fractions of ATP or NADPH that are exchanged between plastid and cytosol through ATP/ADP translocator (T.ATP) and malate/oxaloacetate shuttle (T.NADPH) depend on the FQR/FNR ratio (the ratio between cyclic [FQR] and non-cyclic [FNR] electron flow through photosynthesis light reactions). Cyclic electron flow through photosynthesis light reactions increases ATP yield without corresponding increase in NADPH formation. This estimate was done under assumption of fixed flux ratio photorespiration / photosynthesis = 0.25. Positive values of T.ATP/ATP indicate import of ATP to the plastid from cytosol, zero value indicate self-sufficient ATP balance in the plastid, while the negative values points on ATP export to cytosol. Negative values of T.NADPH/NADPH indicate export reduced equivalents from plastid via malate/oxaloacetate shuttle, while zero values indicate self-sufficient NADPH balance in the plastid. The shaded area, an ATP/NADPH ratio of 1.3 - 1.5 indicates the ratio required to ensure CO_2_-fixation in the Calvin-Benson-Cycle [63, 73]

**Fig. 7:**
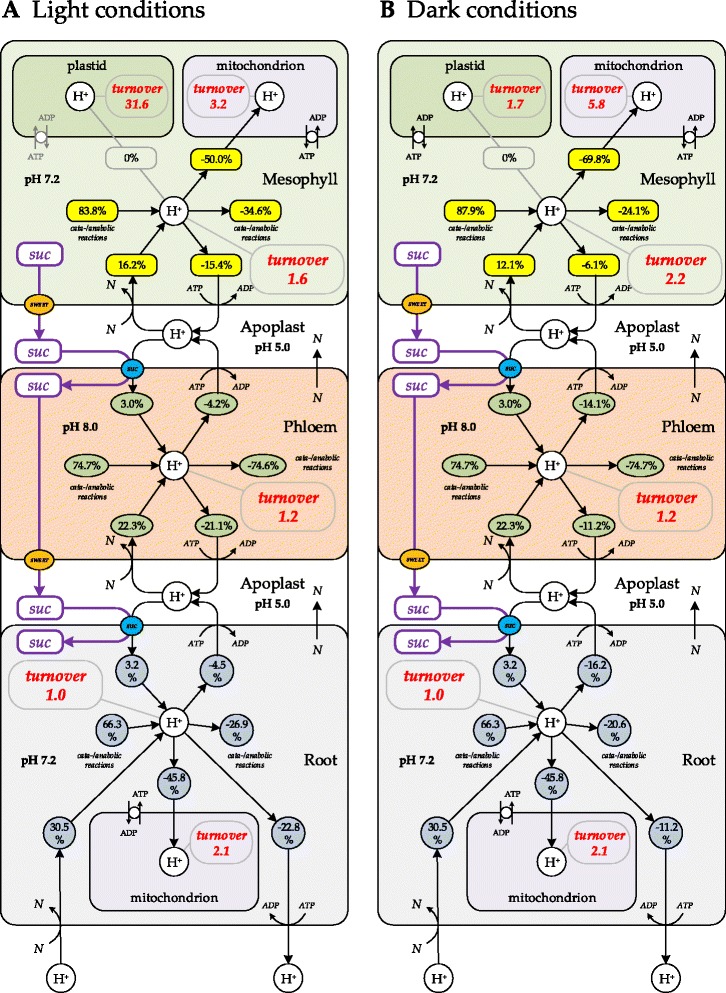
Proton balance in relation with sucrose translocation and growth conditions. The proton balance was predicted by FBA in all three compartments of the model: mesophyll, phloem and root. The presented ‘light’ conditions are: photorespiration/photosynthesis = 0.25 and FQR/FNR = 0.37. Under these constraints the ATP balance in plastid is predicted to be self-sufficient (Fig. 6), therefore there was no ATP and H^+^ exchange between plastid and cytosol. The contribution of major H^+^-producing/consuming processes into overall proton balance in each compartment was summarized and denoted as percentages of contribution. Different shapes and colours of the nodes represent the different pools of protons. The proton turnovers in each compartment were normalized per cytoplasm of root, since it was almost invariant under both light conditions*.* The ‘root’ compartment exchanged protons with the environment, which were acquired in symport with the nutrients and excreted via H^+^/ATPase. *N* – nutrients

Thus, using FBA with different constraints, the contribution of photorespiration, cyclic (FQR) and non-cyclic (FNR) electron flow during photosynthesis were scouted while monitoring ATP/NADPH ratio in the plastid (Table 3 and Fig. 6). The positive value of ATP transport (T.ATP/ATP turnover) through nucleoside triphosphate transporters (NTT) [61] under low FQR/FNR values indicated an insufficient ATP synthetic capacity of plastid and a corresponding need to import ATP from the cytoplasm to fulfil the requirements for carbon fixation. In turn, the negative values of ATP transport related to an overproduction of ATP in the plastid and a corresponding necessity to export it to the cytoplasm. Thus, by balancing levels of photorespiration and cyclic electron flow through photosynthesis light reactions plants are able to fine-tune the ATP/NADPH capacity and to optimize CO_2_ fixation under giving growth conditions. The FBA in light conditions under assumption of a ratio of photorespiration to photosynthesis of 0.25 [53] and a ratio of cyclic to non-cyclic electron flow through photosynthesis light reactions of 0.37 resulted in a self-sufficient ATP balance in plastid (ATP exchange with cytoplasm = 0; Fig. 6). These values (photorespiration/photosynthesis = 0.25; FQR/FNR = 0.37) were used to visualize the predicted proton fluxes in the light condition (Fig. 7) and as standard conditions for further modelling. From Fig. 6 we concluded that under experimentally known ATP/NADPH ratios of 1.3 - 1.5 [1, 63, 73] it is very likely for the plastid to import ATP from the cytoplasm and to export reduced equivalents via malate/oxaloacetate shuttle (Fig. 5).

Redox-potentials are required for nitrogen- and sulphur-reduction pathways and are provided in form of reduced ferredoxin, either by PLR under the light or by PPP in the dark. In the model under light conditions, activity of these pathways withdrew electron flux and correspondingly reduced overall NADPH yield of photosynthesis. NADPH formed by ferredoxin-NADP-reductase was mainly utilized by the Calvin-Benson cycle in order to drive CO_2_ fixation and to form triose phosphates. The excess of NADPH was diverted from plastid to mitochondrion via malate/oxaloacetate shuttle [64]. In mitochondria, the exported reduced equivalents were used for ATP formation via oxidative phosphorylation (Fig. 5). According to FBA, in the dark, the plastid consumed imported GAP from the cytoplasm to provide carbon flux through the CBC and further to oxidative part of PPP in order to generate NADPH required for reductive processes of the CBC itself, as well as for N- and S-reduction. A by-product of the oxidative part of PPP was CO_2_, which was partially utilized in CBC itself, but was also excreted from the plastid.

The ATP synthesis in the plastid by ATP synthase (with stoichiometry *H*_*in*_^+^/*ATP* = 4.0 [57, 58]), was driven by the *H*_*in*_^+^ − motive force between thylakoid lumen and the plastid stroma (Fig. 5). Therefore, addition of cyclic electron transfer by ferredoxin-plastoquinone reductase (FQR) to the photosynthetic light reactions contribute to an increase in ATP yield without corresponding NADPH yield [55, 56]. Additionally, we also investigated potential impact of *H*_*in*_^+^ − leakage from lumen back to stroma, which increases *H*_*in*_^+^/*ATP* stoichiometry. As it appears, within the framework of this model, it was impossible to provide reliable constraints to estimate the contribution of this process, therefore this process was eliminated. Overall, the FBA of the current form of the model predicted that ATP can be imported or exported from/to the cytosol to fulfil the ATP balance in photosynthetically active plastids. The overall balance of ATP in the plastid was regulated by levering photorespiration and cyclic electron flow through the photosynthesis light reactions.

In fact, mitochondrial oxidative phosphorylation has a greater capacity for ATP synthesis than plastid photophosphorylation, producing 3 ATP per NADH compared to the 1.5 ATP per NADPH in plastid [63, 73, 74]. Additionally, the activity of the mitochondrial ATP/ADP translocator is much higher than the activity of the plastidial ATP/ADP translocator [63, 66]. Therefore, it seems logic to conclude that ATP in the cytoplasm is more efficiently provided by the mitochondrion than potentially by the plastid. The yields of ATP produced per NADPH predicted by the model in different compartments are in line with the published data (Table 4).

**Table 4:** Comparison of metabolite turnover predicted by FBA with known values for *Arabidopsis thaliana*

Ratio of precursor turnover [mol i/mol j]	Compartment	This model					Published values	[Ref]
		Dark	Light					
			FQR ^f)^ = 0 PR ^e)^ = 0.25	FQR / FNR ^f)^ = 0.37 PR ^e)^ = 0.25	FQR / FNR ^f)^ = 0.5 PR ^e)^ = 0.25	FQR / FNR ^f)^ = 0.1 PR ^e)^ = 0		
Photon/CO_2_	mesophyll	-	16.13	17.12	18.16	11.96	9.55	[27]
Photon/*NO*_3_^−^	mesophyll	-	104.37	110.71	117.46	77.38	75.87	[27]
CO_2_/*NO*_3_^−^	mesophyll	-	6.47	6.47	6.47	6.48	7.95	[27]
P/O	mesophyll mitochondrion	4.62	4.99	4.98	4.58	4.99	2.5 – 5	[75]
T.ATP/ATP ^a)^	plastid	1	+0.178 [import]	0	0	+0.083 [import]		
NADP^+^-MDH/NADPH ^b)^	plastid	0	−0.147 [export]	−0.063 [export]	0	−0.095 [export]		
T.H^+^/H^+^^c)^	plastid	0	−0.050 [export]	0	0	−0.002 [export]		
ATP/NADPH	plastid	0.35	1.44	1.57	1.68	1.42	1.3 – 1.5	[63, 73]
ATP/NADH	mesophyll mitochondria	2.63	2.50	2.49	2.64	2.49	up to 3	[74]
*H*_*in*_^+^/ATP	plastid	-	4.0	4.0	4.0	4.0	4	[57, 58]
RQ ^d)^	plant	1.83	-	-	-	-	0.8-1.6	[63]
Metabolite turnover [mol i/mol X]								
ATP (× 10^11^)	plastid	0.31	8.04	7.94	8.70	5.53		
NADPH (× 10^11^)	plastid	0.87	5.59	5.04	5.18	3.89		
H^+^-turnover (× 10^12^)	plastid	0.17	2.75	3.15	3.45	2.05		

The turnover are quantified for different light conditions (dark vs. light) and different involvement of cyclic electron flow through photosynthesis system assuming fixed degree of photorespiration (either 20 % of 0 % of photosynthesis)

FQR - ferredoxin-plastoquinone reductase, flux through FQR is the cyclic electron flow through the photosynthesis light reactions; FNR - ferredoxin-NADP^+^-oxidoreductase (EC 1.18.1.2), flux through FNR is the non-cyclic electron flow through the photosynthesis; NADP^+^-MDH – NADP^+^-dependent malate dehydrogenase (EC 1.1.1.82), part of malate/oxaloacetate shuttle, which translocate excess of reduced equivalent to mitochondria; photoresp. – photorespiration; X – biomass

^a)^ – part of ATP from the total ATP turnover in plastid, which is exchanged with cytoplasm through ATP/ADP translocator

^b)^– part of NADPH from total NADPH turnover in plastid, which is exchanged with cytoplasm through malate/oxaloacetate shuttle

^c)^– part of H^+^ from the total H^+^ turnover in plastid, which is exchanged with cytoplasm through transporter

^d)^– respiratory quotient

^e)^– photorespiration/photosynthesis ratio, constrain PR = 0.25 is the ratio between flux through photorespiration and photosynthesis, particularly through RuBisCo in Calvin-Benson cycle

^f)^– FQR/FNR ratio represents ratio between cyclic and non-cyclic electron flows in photosynthesis light reactions

The efficiency of the photosynthetic reactions could be evaluated based on the precursor ratios (Table 4). For example, the achieved *hv*/CO_2_ ratio in the model was around 16, which was close to the published *hv*/CO_2_ ratio of 9.55 for *Arabidopsis thaliana* [27]. Also other metabolic indicators were predicted to be within the range of published values (Table 4), suggesting that despite the limitations outlined above, in general the stoichiometry of the plastid metabolism was correctly presented.

#### Mitochondrion

The mitochondrion in our model contained only two pathways, the TCA cycle and the oxidative phosphorylation. All intermediates of TCA cycle were exchangeable with the cytoplasm, except succinyl-CoA, which was a part of the mitochondrial conserved moiety CoA/acetyl-CoA/succinyl-CoA. Therefore, in the ‘light’ scenario the reactions of NAD-dependent isocitrate dehydrogenase (EC 1.1.1.41), 2-oxoglutarate dehydrogenase and succinate-CoA ligase (EC 6.2.1.5) were inactive, since 2-oxoglutarate was exchanged into the cytoplasm and through anaplerotic reactions was converted into succinate, which returned into the TCA cycle bypassing the reactions of NAD-dependent isocitrate dehydrogenase, 2-oxoglutarate dehydrogenase, and succinate-CoA ligase.

However, in the ‘dark’ scenario, these reactions were active due to differentially loaded glycolysis and consequently different input with primary metabolites into the anaplerotic reactions. The TCA cycle produced its own NADH, but also accepted cumulative redox-potential translocated into the mitochondrion through the malate/oxaloacetate shuttle from the plastid and cytoplasm (Fig. 5). Thus, in the light, the mitochondrial MDH produced 99 % of the NADH (9 % due to own TCA flux and 91 % due to import of L-malate from the cytoplasm), pyruvate dehydrogenase produced 1 % of mitochondrial NADH, and NAD-dependent isocitrate dehydrogenase was predicted to be inactive. CO_2_ production by mitochondria was very low in the light conditions due to inactivity of NAD-dependent isocitrate dehydrogenase and 2-oxoglutarate dehydrogenase, although the ATP production was quite high. Consequently, the predicted ATP/CO_2_ ratio was 65.9. In the dark, the NADH balance was complemented by activities of all reactions of the TCA cycle and correspondingly CO_2_ production was high leading to an ATP/CO_2_ ratio of 6.

In the model, all formed NADH in mitochondrion was consumed by complex I of the oxidative phosphorylation. There were no assigned ATP-consuming reactions in the mitochondrion, therefore all formed ATP from complex V of the oxidative phosphorylation was exported to the cytoplasm. The efficiency of oxidative phosphorylation was assessed by the amount of ATP formed per amount of oxygen consumed (P/O ratio). In reality, the P/O ratio is a variable parameter due to substrate availability and proton leakage. The theoretical value of P/O is 5.0, but practically measured values are generally greater than 2.5 [75]. The FBA analysis suggested that the P/O ratio of the model was greater than 4.6 in both mesophyll and root mitochondria and in both ‘light’ and ‘dark’ conditions (Table 4). Thus, we assume that the balances of redox and energy reactions accepted in the sub-compartment ‘mitochondria’ of the model depict biological reality.

#### Cytoplasm

In general, the ATP pool in the mesophyll cytoplasm can be replenished from three potential sources: (i) glycolysis in the cytoplasm, (ii) oxidative phosphorylation in the mitochondrion and (iii) ATP synthase in the plastid. The FBA of our model predicted that in light conditions (in case of FQR/FNR = 0.37; photorespiration = 0.25) 83.2 % of the cytoplasmic ATP turnover was supplied from mitochondrial metabolism and 16.8 % was provided by glycolysis and other cytoplasmic processes (Table 5). Under dark conditions, the glycolytic contribution to the ATP turnover in cytoplasm became 15.7 % and export of ATP from mitochondrion correspondingly increased to 84.3 %. Although, in dark, about 18.2 % of the ATP gained in the cytoplasm was translocated into plastid and the rest was used for fuelling metabolic reactions (Table 5). Overall, the relative ATP turnover [*mol ATP*/*mol X*] was higher in the dark condition than in the light condition (Table 5), but ATP producing capacity of mitochondria remained similar. This difference in ATP turnover resulted from higher glycolytic activity due to higher glucose metabolism in the dark (e.g. starch degradation). Nevertheless, the major allocations of ATP for growth and non-growth associated processes predicted by the FBA in this model were in line with previously reported values for *Arabidopsis thaliana* [33].

**Table 5:** Major ATP producing/consuming processes in cytoplasm of the mesophyll quantified by FBA

Major ATP …	‘Light’ FQR / FNR = 0.37 photoresp. = 0.25	‘Dark’
Producing processes	83.2 % import from mitochondrion 16.8 % by glycolysis and other processes	84.3 % import from mitochondrion 15.7 % by glycolysis and other processes
Consuming processes	74.4 % by cata-/anabolic reactions 25.6 % by H^+^-ATPase	74.1 % by cata-/anabolic reactions 7.7 % by H^+^-ATPase 18.2 % export to plastid
Relative ATP turnover [*mol ATP*/*mol X*]	9.644e10	1.675e11

The non-growth associated ATP expenses of the plasma membrane H^+^-ATPase can be considered as maintenance. According to the FBA, the plasma membrane H^+^-ATPase consumed 25.6 % of the cytoplasmic ATP in light conditions (in case of FQR/FNR = 0.37, photorespiration = 0.25) and only 7.7 % of the cytoplasmic ATP in dark conditions (Table 5).

The NADH balance in the cytoplasm was comprised by major producing reactions (anplerotic reactions, glycolysis, C1-metabolism, pectin biosynthesis, nucleotides and amino acids biosynthesis, photorespiration) and consuming (cytoplasmic nitrate reduction, C1-metabolism and photorespiration in light conditions) reactions. The FBA of the model predicted active export of reduced equivalents from the plastid to the mitochondrion via the malate/oxaloacetate shuttle in form of malate and oxaloacetate during light conditions (Fig. 5). However, in light conditions photorespiration (particularly hydroxypyruvate reductase) became an additional NADH sink. In the dark, there was no export of reduced equivalents from the plastid, but additionally relatively higher glycolytic flux provided a surplus of NADH, which was translocated into the mitochondrion via the malate/oxaloacetate shuttle to be converted into ATP (Fig. 5). Thus, in the light, the mitochondrion was the sink for excess reduced equivalents formed in the plastid during photosynthesis, and photorespiration was additional sink for cytoplasmatic NADH. In the dark, the mitochondrion was the only sink for excess (up to 35 %) of NADH formed in the cytosol by relatively high glycolytic flux. At the same time, the mitochondrion was the major ATP supplier for cytoplasmic reactions both in light and dark growth conditions.

### Proton balance & sucrose translocation

The main objective of this model was the reconstruction of the proton-fluxes created by the activities of the redox and energy metabolism under different light conditions and their relationship with the translocation of sucrose. The model accounted for main metabolic processes (e.g. reactions and exchange transport) that participated in proton turnover in all compartments and sub-compartments (Table 6). For example, in the model, in the mesophyll cytoplasm are 40 proton-producing processes (reactions and transporters) and 18 proton-utilizing processes.

**Table 6:** Numbers of H^+^-exchanging processes (reactions and transport) accounted for in the model

	H^+^-exchanging processes		
Compartment	Producing	Consuming	
Mesophyll cytoplasm	40	18	
Mesophyll plastid	4	7	
Mesophyll mitochondrion	4	3	
Phloem	8	5	
Root cytoplasm	40	21	
Root mitochondrion	4	3	
Apoplast un-/loading	4	8	
Exchange with env.	4	4	
In total:	108	69	177

In the model, protons entered *Arabidopsis thaliana* from the environment in symport with nutrients into the root (e.g. nitrate, sulphate, phosphate; Fig. 4 and Additional file 1: Figure S1). However, the amount of protons entered in that way exceeds the amount protons going to transport the nutrients, therefore the excess of protons was excreted through H^+^-ATPase back to environment (Fig. 7) resulting in overall consumption of protons by the plant and accompanied alkalinisation of the growth medium. In the model, the efflux of protons from compartments was carried out by the plasma membrane H^+^-ATPase (Fig. 7). The turnover of protons predicted by the FBA in each compartment was normalized relative to the turnover of protons in the root cytoplasm (Fig. 7). The metabolic activity and the proton turnover of the root compartment in the model remained almost invariant under light and dark conditions and therefore this compartment was selected as a reference point for data normalization.

In both growing tissues (mesophyll and root), the major consumer of cytoplasmic protons was the mitochondrion (Fig. 7). For example, mesophyll mitochondria consumed 50 % of cytoplasmic protons under light growth conditions (in case of FQR/FNR = 0.37, photorespiration = 0.25). In mitochondria, major proton utilizing processes were oxygen reduction in oxidative phosphorylation. Thus, in order to maintain trans-membrane proton gradients, mitochondria constantly needed additional proton input, which came from the cytoplasm.

Also many metabolic processes resulted in proton production/utilization and therefore additionally contributed to the proton balance in the cytoplasm. Particularly in the light (in case of FQR/FNR = 0.37, photorespiration = 0.25), the plastid provided GAP, which entered glycolysis downstream of sugar phosphorylation reactions, which are producing substantial amounts of protons (Fig. 7). In the plastid, under light conditions, ATP synthase and photosynthesis light reactions produced a surplus of protons due to water photolysis, which was completely utilized by the CBC and nitrogen/sulphur reduction. Thus, turnover of proton in the photosynthetically active plastid was very high relative to the other compartments (Fig. 7), and the plastdal proton balance is self-sufficient in this conditions (FQR/FNR = 0.37, photorespiration = 0.25).

Under dark conditions, starch was decomposed to glucose, which involved phosphorylation by glucokinase (GLK; EC 2.7.1.2) and a release of protons contributing to approximately 17 % of the cytoplasmatic proton pool. This proton excess was mainly utilized by the fully functional TCA cycle in mitochondria. Correspondingly, the mesophyll in dark conditions pumped less protons to the apoplast (Fig. 7). The overall proton consumption by mitochondria was higher in dark than in light growth conditions (Fig. 7, Table 3).

FBA of proton fluxes allowed quantifying the net-fluxes of protons from phloem to mesophyll and root. Further, taking in account pH differences between compartments, the protons enter the root cytoplasm from the phloem along their concentration gradient allowing symport of solutes including sucrose via SUC and STP transporters. The sucrose flux to the root (as the sink tissue) was in accordance with proton symport mechanisms of SUCs and STPs and the proton balance of the tissues involved (Figs. 3, 4 and 7). Furthermore, the directions of proton fluxes between mesophyll and phloem did not disturb sucrose efflux from the mesophyll to the phloem due to independence of the SWEET transporters from the proton motive force. Under both scenarios (‘light’ and ‘dark’) the proton fluxes estimated by the FBA (Fig. 7) matched specific requirements by the molecular mechanisms of sucrose transporters (SWEET and SUC, STP) in the different tissues (Figs. 3, 4 and 7).

## Conclusions

In this work we present a multi-compartmental metabolic model of growing *Arabidopsis thaliana*. The flux balance analysis (FBA) of the model quantified sugar metabolism, central carbon metabolism, photosynthesis, energy and redox metabolism, proton turnover, sucrose translocation from mesophyll to root and biomass growth under both dark- and light-growth conditions with corresponding growth either on CO_2_ (in the light) or on starch (in darkness). The model showed that in light conditions, interplay between photorespiration and photosynthesis including both cyclic and non-cyclic electron flow defined the ATP balance of the plastid. The plastid was found to be either deficient, self-supported or producing a surplus of ATP. The excess of redox potential from the photosynthetic light system was translocated to the mitochondrion via the malate/oxaloacetate shuttle. Thus, photosynthetically active plastids could achieve the ATP/NADPH ratio required for CO_2_ fixation. Also, the FBA predicted that the mitochondria were the main ATP provider for cytoplasmic processes together with glycolysis under both light- and dark-growth conditions. At the same time, the mitochondria were the main sink for reduced equivalents translocated both from plastid and from cytoplasm as well as for the protons.

The model described all main metabolic processes participating in the proton metabolism. The translocation of sucrose among plant tissues was associated with an integral balance of protons, which in turn was partially defined by operational modes of the energy metabolism (photosynthesis, respiration). The proton fluxes predicted by FBA generally corresponded to the molecular mechanisms and functional peculiarity of the sucrose transporters SWEET and SUC/STP proton-symporters and net-flux of sucrose from source to sink tissue. Thus, our multi-compartmental model adequately described the growth stoichiometry and sucrose translocation processes of *Arabidopsis thaliana* in light and dark conditions. The FBA was used to optimize the network structure and to prepare it for further analysis of the distribution of the intracellular fluxes based on experimental measurements by Metabolic Flux Analysis (MFA).

## Methods

### Network reconstruction

The primary information on the metabolic reactions regarding stoichiometry, direction, metabolite elemental formula, charge state, and associated genes was collected from AraCyc [48] using the Pathway Tool v19.0 [76] and additionally were verified with TAIR [77, 78], KEGG [79] and ChEBI [80] databases.

The model reconstruction was performed with Insilico Discovery™ (Insilico Biotechnology AG, Stuttgart, Germany). The structured metabolic network of *Arabidopsis thaliana* was reconstructed based on the decision to take in account only those metabolic processes, which contributed to better understanding of sugar metabolism, central carbon metabolism, energy metabolism, proton turnover, biomass growth and sucrose translocation among tissues in *Arabidopsis thaliana*. Thus, metabolic reactions from the following pathways were used in the reconstructed network: 2-oxoglutorate decarboxylation to succinyl-CoA; adenosine nucleotides *de novo* biosynthesis; amino acids biosynthesis; aspartate degradation; Calvin-Beson cycle; chorismate biosynthesis; fatty acids biosynthesis; folate metabolism; glutamine biosynthesis; glycolysis; homoserine biosynthesis; inosine-5’-phosphate biosynthesis; maintenance; malate/oxaloacetate shuttle; nitrate reduction; ornithine biosynthesis; oxidative phosphorylation; PRPP biosynthesis; pentose-phosphate pathway; phosphorus metabolism; photosynthesis light reactions; photorespiration; purine nucleotides *de novo* biosynthesis; pyrimidine ribonucleotides interconversions; pyruvate decarboxylation to acetyl-CoA; pyruvate fermentation; starch biosynthesis; starch degradation; sucrose biosynthesis; sucrose degradation; sulphate reduction; TCA cycle; UDP-glucose biosynthesis; UDP-glucoronate biosynthesis; uridine-5’-phospahte biosynthesis. Many biosynthetic reactions concatenated in linear pathways (e.g. nucleotide or amino acid biosynthesis) were lumped in order to reduce model complexity. All reactions in the model were manually transferred from the database, and manually curated.

The biomass was assumed to be composed of the following polymers: proteins, lipids, RNA, carbohydrates (i.e. starch, sucrose, cellulose, pectin) and ash [33, 40]. The amino acids composition of a protein was accepted from literature [33]. The lipid composition was assumed to be similar to yeast one and considered as a polymer of 9.6 % hexadecanoate, 20.6 % *cis*-octadecenoate, 34.7 % *cis*-hexadecenoate, 2.4 % octadecanoate, 0.006 % Linoleate and 32.4 % glycerol. RNA composition was derived by reversed sequence analysis of protein amino acid sequences in FASTA format available from TAIR: 32 % A, 26 % U, 9 % C and 33 % G. Based on these data, the biomass macromolecular composition was considered to be (w/w) 20 % cellulose, 20 % pectin, 10 % starch, 12 % sucrose, 25 % proteins, 9 % lipids, 1 % RNA and 3 % ash. The mass ratio between shoot and root was accepted as 85/15 (w/w) based on own measurements of fresh weight of 21 days old plants grown under long-day (16 h light) conditions in hydroponic culture.

### Compartmentalization

In accordance with concept of sucrose emitting tissues and sucrose demanding tissues as well as under consideration of the molecular mechanism of sucrose translocation mechanisms (Fig. 3), under different energy producing modes (‘light’ and ‘dark’) we generalized the following compartments: a super-compartment ‘plant’ which included (i) the autotrophic sub-compartment ‘mesophyll’ with sub-compartments ‘plastid’ and ‘mitochondrion’, (ii) the heterotrophic sub-compartment ‘root’ with only one sub-compartment ‘mitochondrion’ and (iii) non-growing transport compartment ‘phloem’; whereas the inner space of the super-compartment ‘plant’ served as the extracellular compartment ‘apoplast’. Sub-compartment ‘plastid’ was required to separate photosynthetic light reactions, sulphate and nitrite reduction, Calvin-Benson cycle with associated triose phosphates, hexose phosphates, NADPH/NADP moiety, ATP synthesis and starch synthesis (Additional file 1: Figure S1). Sub-compartment ‘mitochondrion’ was required to separate TCA cycle and associated with its activity NADH/NAD moiety and ATP synthesis (Additional file 1: Figure S1). Compartmentalization was required in order to separate pools of similar metabolites between compartments with different metabolic specificities and to orchestrate their exchange through transport steps [33]. Correspondingly, ‘mesophyll’ and ‘root’ were considered growing compartments, whereas ‘phloem’ was assumed a non-growing connecting compartment. The exchange processes between tissues were routed through the apoplast.

### Model annotation

In the model, each biochemical reaction (classified with E.C. number) was referred to corresponding KEGG reaction (KeggID). Additionally, reactions were annotated with accession numbers of genes whose products perform this biochemical process (GeneID). In case of lumped reactions, all corresponding GeneIDs were associated with lumped reaction. This annotation was later used to match the model with mass-spectrometry based proteomic data [81] and to validate the presence of reactions in the model.

The final version of the model was exported in SBML and MATLAB format (Supplemental data) as well as the stoichiometric matrix of the model and high-resolution printout (JPG) of the model with all marked reactions. Corresponding databases of the compounds/metabolites, transformers/reactions and genes used in the model are provided in in ASCII format (Supplemental data).

### Model assumptions and biologically based constraints

In order to reduce the complexity in the description of processes within the multi-tissue plant and to focus mainly on the mechanisms of sucrose translocation, the model was formulated under the following assumptions:

1. Sucrose was considered as the only carbohydrate that is exchanged among tissues.
2. The anatomy of the whole plant was reduced down only to three tissues: autotrophic mesophyll, heterotrophic root, interconnected by transport phloem. The model was restricted to the mesophyll as always being the ‘source’ tissue of sucrose both in light conditions (during photosynthesis) and in dark conditions (non-photosynthetic condition, carbon derived from starch). The root was always defined as the ‘sink’ tissue for sucrose. The root took up sucrose and used it for biomass formation, but did not store it with a purpose to emit it back to phloem. Thus, we have consciously constrained the exchange of sucrose to a unidirectional net-flux from autotrophic mesophyll to heterotrophic root (Figs. 3 and 4).
3. Sucrose was considered to be also a part of the formed biomass. Nevertheless, we did not consider the possibility that deposited sucrose can be returned to sucrose pool into the phloem for its exchange. Thus, sucrose, which was directed to biomass, was not considered as available for growth or exchange through the free sucrose pool.
4. Starch was considered as a metabolite that can be formed and deposited during light growth phase and used as the carbon and energy source during dark growth phase. We assumed that starch was accumulated only in autotrophic tissues (mesophyll), but not in heterotrophic tissues (root).
5. Starch was formed only during the light growth phase, and stored as a part of biomass. It was not degraded even partially during light growth phase.
6. Starch was considered as the only carbon source for growth of the plant during dark conditions.
7. In this version of the model, we did not consider exchange of amino acids and organic acids (glutamate, acetic, citric acids) between tissues.
8. Fatty acid biosynthesis was considered to be cytoplasmic to reduce complexity of redox balance in plastid.
9. ATP/ADP was made exchangeable between compartments (Fig. 4) through the adenine nucleotide translocator.
10. NAD(P)H/NAD(P) could not be exchanged between compartments. However, the reduced equivalent could be transferred between compartments through malate/oxaloacetate shuttle.
11. Phloem played only a role in transport processes and deliver nutrients and water from root to mesophyll, and sucrose from mesophyll to root as unidirectional net-fluxes (Fig. 4).
12. Nutrient uptake (nitrate, orthophosphate and sulphate) followed a proton-symport mechanism.
13. Proton transport against know pH gradient was always active (ATP-dependent), unlike transport along known pH gradients, which was considered as being passive (Figs. 3 and 4).
14. The only exchangeable metabolites from central carbon metabolism between plastid and cytoplasm in the mesophyll were D-glyceraldehyde 3-phosphate (GAP) and maltose. Other potentially exchangeable metabolites (i.e. other triose phosphates, hexose phosphates) were not considered in the model to reduce the complexity.
15. Gas exchange between plant and environment was considered as being passive.

### Network setup

Before proceeding to advanced network analysis, the detailed consistency check of the network stoichiometry through elemental and charge balance analysis was performed with Insilico Discovery™ (Insilico Biotechnology AG, Stuttgart, Germany). In addition to known reaction stoichiometry, this analysis relied on known elemental composition and charge (at specific pH) of metabolites. Consistency check of elemental and charge balances allowed identifying and revising/eliminating: (i) compounds for which elemental composition and charge were unknown and (ii) inconsistent reactions, polymerizations and transport steps with unbalanced elements or charges. A Check for dead-ends and unused transformers allowed identifying and eliminating transformers and network regions which were not capable of operating at steady state. A check for irreversible transformers allowed visualizing and approving those transformers, which were irreversible from thermodynamic point of view. Irreversibility of a transformer was important constraint for the Flux Balance Analysis.

### Topological analysis of the network

Topological analysis of the stoichiometric matrix **S** of the network is very powerful tool in optimization of the metabolic network structure, because it identifies and illustrates how substances flows within a network. This analysis also allowed identification of false or/and unused network structures. The *degree of freedom* estimated the dimensions of the network, and further requirements to solve it with experimentally derived measurements. The lower degree of freedom, the easier solutions can be found. Analysis of *conserved moiety* revealed sets of compounds, whose sum always remained the same even under dynamic conditions. The number of conserved moieties equalled the number of linearly dependent balance equations. Analysis of conserved moieties can reveal false-positive conserved moieties which had accidentally appeared due to designed network structure. Accidental false-positive conserved moieties were eliminated and only biologically relevant conserved moieties (e.g. NAD(P)H/NAD(P), acetyl-CoA/CoA/succinyl-CoA) were remain in the network. Analysis of *blocked transformers* identified regions of the network, which were not capable of operating at steady state due to the irreversibility constraints of the transformer equation. Computation of the base vector of the *nullspace* revealed “functional” and “non-functional” regions of a metabolic network. Nullspace of **S** is a set of all solutions of the linear equation system **S** × **v** = **0**. **S** was the stoichiometric matrix of the network (Supplemental data) and **v** was the rate vector of metabolic fluxes. Computation of *parallel routes and cycles* revealed a set of reactions/transporters/polymerizators which (i) were capable of maintaining a steady state, (ii) could not be decomposed, and (iii) in total did not consume or produce an external substrate/product. From condition (iii) followed that the net reaction of parallel routes and reaction cycles was zero. In the complex networks, there was a high chance for the accidental formation of parallel routes and cycles which increased internal degree of freedom. Parallel routes and cycles substantially increase the number of elementary flux modes without leading to new phenotypic behaviour. Therefore, their number had to be minimized in order to use elementary mode analysis for evaluation of maximal product yield. Computation of *elementary modes* revealed a unique set of smallest sub-networks that allowed a reconstructed network to function in steady state. Elementary mode analysis took into account stoichiometry and thermodynamics when evaluating whether a particular metabolic route or (sub-)network was feasible and likely for a given set of proteins/enzymes. The topological analysis of the model was performed with Insilico Discovery™ (Insilico Biotechnology AG, Stuttgart, Germany).

### Flux Balance Analysis (FBA)

The distribution of intracellular metabolic fluxes through the metabolic network was investigated under pseudo steady state assumption (**S** × **v** = **0**) using constraint-based Flux Balance Analysis (FBA). In order to calculate metabolic flux distribution, maximization of biomass formation was formulated as objective function. The FBA provided a method for computing flux distributions in the metabolic networks by linear optimization of the objective function:

$$ \begin{array}{l}\mathrm{maximize}\kern0.75em \mathbf{Z}={\mathbf{c}}^{\mathbf{T}}\mathbf{v}\\ {}\mathrm{subject}\ \mathrm{t}\mathrm{o}\kern0.75em \mathbf{S}\times \mathbf{v}=\mathbf{0}\\ {}\mathrm{and}\kern3em {\nu}_i^{\min}\le {v}_i\le {\nu}_i^{\max}\end{array} $$

where **Z** was the objective function, **c** was a vector of weighting factors, *v*_*i*_ was the *i*th element of ***v*** and *ν*_*i*_^min^ and *ν*_*i*_^max^ were the minimum and maximum constraints on *v*_*i*_. The topology of stoichiometric matrix (**S**), together with lower (*ν*_*i*_^min^) and upper (*ν*_*i*_^max^) boundaries for the vector of metabolic fluxes (*v*), in combination with thermodynamic properties of reactions (e.g. irreversibility), and steady state condition (**S** × **v** = **0**)provided with sufficient constraints to optimize objective function. The designed metabolic network was subjected to two specific constraints provided in Supplemental data (Table 3). The linear programming problem was solved using Insilico Discovery™ (Insilico Biotechnology AG, Stuttgart, Germany).

## Additional file

Additional file 1: Figure S1. Stoichiometric model of *Arabidopsis thaliana*. The complete stoichiometric model consisted of three tissues-compartments (mesophyll, phloem, root) with two sub-compartments (mitochondrion, plastid). The reconstructed network contained 400 transformers, 413 balanced metabolites and 742 ORFs associated with corresponding reactions. The image was generated with Insilico Discovery™ (Insilico Biotechnology AG, Stuttgart, Germany). (JPG 18396 kb)

## Metabolites

3PGA: D-glycerate 3-phosphate (ChEBI:58272)
DHAP: Dihydroxyacetone phosphate (ChEBI:57642)
f6p: Fructose-6-phosphate (ChEBI:57579)
g6p: Glucose-6-phosphate (ChEBI:58247)
GAP: D-glyceraldehyde 3-phosphate (ChEBI:59776)
H^+^: Proton (ChEBI:24636)
*hv*: Light photon (ChEBI:302012)
N: is a nitrogen source, in the model it is *NO*_3_^−^
oaa: Oxaloacetate (ChEBI:16452)
P: is a phosphorus source, in the model it is *HPO*_4_^(2−)^, i.e. orthophosphate
P_i_ or *HPO*_4_^(2−)^: Orthophosphate (ChEBI:18367)
S: is a sulphur source, in the model it is *SO*_4_^2 −^
suc: Sucrose (ChEBI:17992)
X: Biomass

## Enzymes

Aldo: Fructose-bisphosphate aldolase (EC 4.1.2.13)
FBP: Fructose 1,6-bisphosphatase (EC 3.1.3.11)
FNR: Ferredoxin-NADP-reductase (EC 1.18.1.2)
FQR: Ferredoxin-plastoquinone reductase
GAPDH: Glyceraldehyde-3-phosphate dehydrogenase (EC 1.2.1.12/13)
GLK: Glucokinase (EC 2.7.1.2)
MDH: Malate dehydrogenase (EC 1.1.1.37)
NTT: ATP/ADP translocator
PFK: 6-phosphofructokinase (EC 2.7.1.11)
PGK: Phosphoglycerate kinase (EC 2.7.2.3)
PGM: Phosphoglucomutase phosphoglucomutase (EC 5.4.2.2)
PRK: Phosphoribulokinase (EC 2.7.1.19)
RPC: Ribulose-bisphosphate carboxylase, RuBisCo (EC 4.1.1.39)
SUC and STP: Sucrose-proton symporters
SWEET: Sucrose efflux transporter (Sugars Will Eventually be Exported Transporters)
TK: Transketolase (EC 2.2.1.1)
TPI: Triosephosphate isomerase (EC 5.3.1.1)
TPT: Triosephosphate/phosphate translocator

## Pathways

CBC: Calvin-Benson cycle
PLR: Photosynthesis light reactions
PPP: Pentose phosphate pathway

## Miscellaneous

g_dw_: Dry weight of biomass [g]
FBA: Flux balance analysis
GO:0015770: Biological process- sucrose transport – The directed movement of sucrose into, out of or within a cell, or between cells by means of some agent such as a transporter or pore
